# Dietary Selenium and β‐Carotene Contribute to the Inverse Association Between Antioxidant Mixtures and Stroke: Evidence From NHANES Using Mixture Models and Machine Learning

**DOI:** 10.1002/fsn3.72442

**Published:** 2026-10-21

**Authors:** Aifang Zhong, Xinmin Deng, Yejun Tan, Rui Lai, Xiaofeng Lv, Xia Zhou, Jiaying Zhu

**Affiliations:** ^1^ Department of Emergency Internal Medicine Guizhou Provincial People's Hospital Guiyang Guizhou China; ^2^ Chengdu University of Traditional Chinese Medicine Chengdu Sichuan China; ^3^ Department of Health Technology and Informatics The Hong Kong Polytechnic University Hong Kong, SAR China

**Keywords:** β‐carotene, dietary antioxidants, joint exposure, machine learning, mixture models, NHANES, selenium, stroke

## Abstract

Traditional single‐factor analyses often fail to comprehensively evaluate the synergistic effects among dietary nutrients. This study aimed to examine the association between combined dietary intake of six antioxidant‐related nutrients and the odds of stroke. Using the NHANES database (*n* = 37,746), we applied 1:1 propensity score matching (PSM) to adjust for baseline confounders, establishing a matched sub‐cohort of 2750 individuals. Targeting six antioxidant‐related nutrients (vitamins A, C, and E, β‐carotene, selenium, and zinc), we used weighted quantile sum (WQS) regression, quantile g‐computation (qgcomp), Bayesian kernel machine regression (BKMR), and four machine learning algorithms to quantify joint exposure effects and estimate the relative contributions of each component. Across these complementary analytical frameworks, higher combined intake of the six nutrients was consistently associated with lower odds of stroke. In the WQS and qgcomp models, selenium and β‐carotene received the largest weights in the observed inverse mixture association. BKMR assigned the highest posterior inclusion probabilities (PIPs) to selenium (0.988) and β‐carotene (0.975), and these two nutrients also ranked as the top two features across all four machine learning algorithms. Non‐linear analyses suggested curvilinear inverse associations for selenium and β‐carotene, with attenuation of the inverse association at higher intake levels. Furthermore, single‐variable effects were parallel across background exposure quantiles, with no statistical evidence of departure from additivity among the six nutrients. In conclusion, higher combined intake of this nutrient mixture was associated with lower odds of stroke. These findings support further investigation of antioxidant nutrients as a dietary mixture rather than as isolated components. Before preventive recommendations can be made, prospective cohort studies with repeated dietary assessment are needed for stroke prevention.

## Graphical abstract

In this NHANES study of 37,746 participants, we examined the joint association of six dietary antioxidant‐related nutrients (vitamins A, C, and E, β‐carotene, selenium, and zinc) with stroke, applying 1:1 propensity score matching (matched sub‐cohort, *n* = 2750), mixture models (WQS, qgcomp, and BKMR), and machine learning with SHAP interpretation. Higher combined intake of these nutrients was associated with lower odds of stroke, and selenium and β‐carotene made the largest and most consistent contributions across these approaches. For both nutrients, the exposure‐response relationship was non‐linear and L‐shaped, with the inverse association leveling off at higher intake levels.

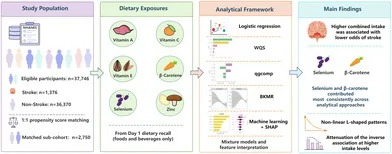

## 1 Introduction

Stroke is a major global public health challenge, ranking as the third leading cause of mortality and the fourth leading cause of disability‐adjusted life‐years (DALYs) worldwide in 2021 (Feigin et al. 2024). With approximately 11.9 million incident cases annually, the global burden is predominantly driven by ischemic stroke (Feigin et al. 2024). Despite continuous advances in acute clinical interventions, the absolute disease burden of stroke continues to increase, largely driven by population aging. Consequently, global stroke‐related DALYs are projected to reach 189 million by 2050 (Feigin et al. 2023). This high incidence is accompanied by a severe prognostic burden, with up to 29% of stroke survivors sustaining permanent disabilities (Kainz et al. 2021). Given the massive prevalent population and the irreversible nature of stroke‐induced disability, strengthening primary prevention strategies is of urgent public health importance. Specifically, optimizing dietary structures—such as adopting a Mediterranean‐style diet—is an important preventive strategy for cardiovascular and cerebrovascular events (Estruch et al. 2018; Lichtenstein et al. 2021).

Consistent with the benefits of overall dietary patterns, longitudinal studies indicate that higher intake of nutrient‐rich foods is associated with a lower risk of cardiovascular and cerebrovascular events (Shan et al. 2020; Mente et al. 2023). In parallel, high intakes of antioxidant vitamins (e.g., vitamins A, C, E, and β‐carotene) and essential trace elements (e.g., selenium and zinc) are inversely associated with stroke risk and adverse prognoses (Mattern et al. 2021; Aune et al. 2018; Li et al. 2025). However, clinical interventions in this domain are complicated by the “antioxidant paradox” (Halliwell 2000): randomized controlled trials (RCTs) evaluating single antioxidant supplements (such as vitamin E or C) have largely failed to demonstrate significant protective effects against stroke. More critically, some trials even indicate an elevated risk of specific adverse cardiovascular and cerebrovascular events (Bjelakovic et al. 2007; Schurks et al. 2010).

The discrepancy between observational findings and clinical trials exposes inherent methodological limitations in traditional epidemiological approaches. First, dietary nutrients do not function in isolation in vivo; rather, they constitute a “whole food matrix” characterized by complex collinearity and interactions (Chen et al. 2023; Townsend et al. 2023). Traditional univariate analytical frameworks fail to adequately capture these synergistic effects, frequently resulting in the overestimation or obscuration of an individual component's true effect. Second, stroke populations within observational cohorts typically exhibit a pronounced clustering of baseline risk factors, such as advanced age and hypertension. In the presence of such systematic baseline differences, conventional linear covariate adjustments often rely heavily on statistical extrapolation, thereby struggling to rigorously eliminate residual confounding (Austin et al. 2021). Furthermore, traditional models cannot adequately characterize non‐linear exposure‐response relationships or potential interactions at the individual level.

To address these methodological challenges, this study aimed to systematically evaluate the associations between mixed exposure to key dietary antioxidants and trace elements and the risk of stroke. By constructing a sub‐cohort via propensity score matching (PSM) to rigorously control for baseline confounding (Austin et al. 2021; Desai and Franklin 2019), and integrating non‐parametric mixture models with machine learning, we sought to quantify the joint association of the antioxidant nutrient matrix with stroke risk while overcoming multicollinearity. Furthermore, we aimed to identify the nutrients that contributed most strongly to the observed mixture association. These analyses may provide epidemiological insight into differences between nutrient intake from mixed dietary sources and isolated antioxidant supplementation, while generating hypotheses for future prospective nutritional studies.

## 2 Materials and Methods

### 2.1 Study Population and Design

This study was based on data from ten consecutive two‐year cycles (1999–2000 through 2017–2018) of the National Health and Nutrition Examination Survey (NHANES), which together comprised 101,316 participants. Stroke history was ascertained with the Medical Conditions Questionnaire item MCQ160F (“Has a doctor or other health professional ever told you that you had a stroke?”), and participants who answered “yes” were classified as having prevalent stroke. Participants were then excluded sequentially for the following reasons: (1) stroke status unavailable (*n* = 46,316), which in most cases reflected the fact that MCQ160F is administered only to participants aged 20 years or older, so that missingness was largely structural among those younger than 20 years rather than a result of item nonresponse; (2) no dietary intake records for the six target antioxidant‐related nutrients (*n* = 10,559), a step that removed the entire 1999–2000 cycle, in which nutrient data were systematically unavailable; and (3) incomplete data on core covariates (*n* = 6695). After these exclusions, 37,746 participants from nine cycles (2001–2002 through 2017–2018) remained eligible, of whom 36,370 had no history of stroke and 1376 reported a history of stroke. The selection process is summarized in Figure 1; unweighted sample sizes by cycle are given in Table S1; and a comparison of included and excluded participants is given in Table S2. Consistent with the age restriction of the stroke item, excluded participants were considerably younger than included participants (mean age, 20.34 vs. 49.30 years).

**FIGURE 1:**
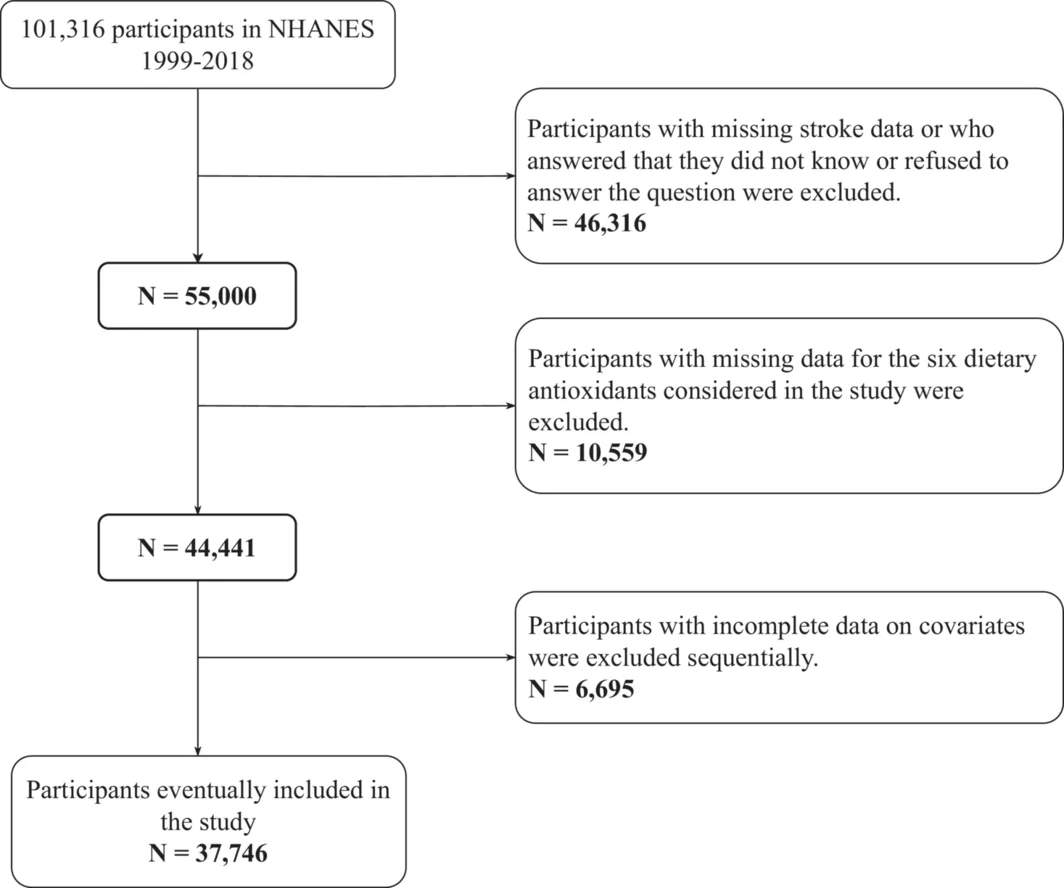
Flowchart of the study population selection from the NHANES database.

### 2.2 Dietary Exposure Assessment and Covariates

Dietary nutrient intakes were assessed using the standardized 24‐h dietary recall interviews from NHANES. To account for data collection variations across different survey cycles, only the first‐day dietary recall data (Day 1) were extracted to ensure a uniform assessment standard; additionally, the nutrient variables evaluated in this study reflect intakes exclusively from foods and beverages, excluding dietary supplements. This study primarily analyzed the mixed exposure to six dietary antioxidants and trace elements: vitamin A, vitamin C, vitamin E, β‐carotene, selenium, and zinc. For statistical analyses, these exposures were treated as continuous variables and also categorized into quartiles (Q1–Q4, with Q1 as the reference group) to evaluate potential non‐linear effects. The models incorporated the following covariates: demographic and socioeconomic characteristics (age, sex, race/ethnicity, education level, and family income‐to‐poverty ratio [PIR]), as well as lifestyle factors and clinical comorbidities (body mass index [BMI], smoking status, drinking status, hypertension, and diabetes).

### 2.3 Propensity Score Matching

Baseline analyses revealed significant imbalances in multiple covariates between the stroke and non‐stroke groups (e.g., age, standardized mean difference [SMD] = 1.100; hypertension prevalence, SMD = 0.895). To control for potential systematic confounding bias, a 1:1 nearest‐neighbor PSM was applied to balance baseline characteristics. The matching quality was evaluated using the SMD. An absolute SMD of < 0.10 was predefined as the threshold for optimal covariate balance (Austin 2009), while an SMD < 0.25 was considered an acceptable limit for residual imbalance, provided it was further adjusted in subsequent multivariable regression models (Rubin 2001; Stuart 2010).

### 2.4 Statistical Analyses

Given that dietary exposures were derived from the first‐day 24‐h dietary recall, the Day 1 dietary sample weight (WTDRD1) was divided by nine to construct a combined weight for the nine pooled 2‐year cycles; this combined weight (WTDRD1/9), together with the survey strata (SDMVSTRA) and primary sampling units (SDMVPSU), was incorporated into the survey‐weighted logistic regression models evaluating the independent effects of individual nutrients. In the full eligible sample, continuous variables were summarized as medians with interquartile ranges and categorical variables as counts and percentages; in the inclusion and covariate‐balance comparisons, means with standard deviations and counts and percentages were used instead, matching the scale on which SMDs were computed. To minimize potential bias arising from extreme baseline imbalances, all evaluations of independent, mixed, and non‐linear effects were conducted exclusively within the matched sub‐cohort generated by PSM (*n* = 2750), except for the baseline comparisons of the original cohort.

Throughout this manuscript, terms such as “joint effect” and “independent effect” refer exclusively to the standard statistical estimators used in mixture modeling literature, rather than causal effects. Similarly, “predictor” and “feature importance” in machine learning analyses denote predictive contribution, not causal relationships.

### 2.5 Independent Effect Evaluation

Within the matched sub‐cohort, multivariable logistic regression models were employed to evaluate the independent associations between individual nutrient intakes and odds of stroke, calculating odds ratios (ORs) and 95% confidence intervals (CIs). A staged (sequential) adjustment strategy was applied: Model 1 served as the unadjusted crude model; Model 2 adjusted for demographic characteristics; and Model 3 further incorporated socioeconomic, lifestyle, and clinical covariates to establish a regression‐adjustment‐following‐matching framework. Linear dose–response relationships were assessed using the *P* for trend test. To account for potential confounding by energy intake, an additional sensitivity analysis further adjusted for total energy intake (kcal/day) as a continuous covariate in the fully adjusted model. As a sensitivity analysis for the quartile‐based analyses, *p* values from trend tests and Q4‐versus‐Q1 contrasts were adjusted using the Benjamini–Hochberg false discovery rate (FDR) procedure.

### 2.6 Mixed Exposure Effect Evaluation

To address the inherent multicollinearity among dietary components, weighted quantile sum (WQS) regression and quantile g‐computation (qgcomp) models were applied to estimate the joint effects of the nutrient mixture. The WQS model estimated the unidirectional overall effect (constrained to the negative direction based on our a priori hypothesis) and the relative contribution weights of each component, whereas the qgcomp model validated the robustness of the joint effect without imposing directional constraints.

### 2.7 Non‐Linear and Interaction Analyses

A Bayesian kernel machine regression (BKMR) framework was implemented to evaluate potential non‐linear relationships and interactions. The independent contribution of each component was quantified by extracting posterior inclusion probabilities (PIPs), and single‐variable effects at varying reference quantiles were extracted to assess potential interactions. Furthermore, to estimate inflection points for clinical interpretability, restricted cubic spline (RCS) and two‐piecewise logistic regression models were additionally fitted within the matched sub‐cohort. These quantitative threshold estimates were subsequently cross‐validated against the non‐parametric BKMR univariate exposure‐response functions.

As sensitivity analyses, the WQS, qgcomp, and BKMR models were additionally adjusted for total energy intake (kcal/day, continuous), with all other specifications identical to the primary models.

### 2.8 Machine Learning and Model Interpretation

To corroborate the results of the regression analyses, four machine learning algorithms—LightGBM, Random Forest, support vector machine (SVM), and XGBoost—were employed to construct predictive models within the matched sub‐cohort. Participants were allocated to a 70% training set and a 30% independent test set using stratified random sampling by stroke status (random seed = 52). Hyperparameters were tuned by five‐fold cross‐validation within the training set to optimize performance and limit overfitting. The analysis was conducted in two stages. First, nutrient‐only models incorporating the six target nutrients were constructed to compare their relative importance. Subsequently, an XGBoost model was developed by integrating the six nutrients with demographic and clinical covariates. Model discrimination in the independent test set was evaluated using the area under the receiver operating characteristic curve (AUC) with a 95% bootstrap confidence interval (CI), accuracy, sensitivity, specificity, and F1 score at a probability threshold of 0.50. Calibration was evaluated using the Brier score with a 95% bootstrap CI and calibration slope. The SHapley Additive exPlanations (SHAP) approach was used for model interpretation. Global feature importance was ranked based on mean absolute SHAP values, and SHAP beeswarm plots were generated to illustrate the non‐linear effects of individual features on model output.

## 3 Results

### 3.1 Covariate Balance Analysis Before PSM

The present study included a total of 36,370 non‐stroke individuals and 1376 participants with a history of stroke (Table S3). Baseline analyses revealed imbalances across multiple core covariates between the two groups (SMDs > 0.10). Specifically, the most substantial imbalance was observed in age (SMD = 1.100), with the stroke group having a higher mean age than the non‐stroke group (65.99 vs. 48.67 years, *p* < 0.001). Regarding clinical comorbidities, the prevalence of hypertension (81.7% vs. 42.0%, SMD = 0.895) and diabetes (35.5% vs. 13.9%, SMD = 0.519) was higher in the stroke group compared to the non‐stroke group (both *p* < 0.001). Furthermore, varying degrees of baseline imbalances were observed in lifestyle and socioeconomic characteristics, including smoking status (SMD = 0.328), PIR (SMD = 0.344), racial composition (SMD = 0.280), education level (SMD = 0.240), and drinking status (SMD = 0.122; all *p* < 0.001; Table S4). Given the baseline imbalances of these covariates within the original cohort, a PSM procedure was implemented to control for potential confounding factors in the subsequent independent and joint exposure analyses.

### 3.2 PSM and Covariate Balance Analysis

The matching procedure paired 1375 participants with a history of stroke with corresponding non‐stroke controls, ultimately yielding a matched sub‐cohort of 2750 individuals (Table S5). Comparisons between the groups before and after matching (Tables S4 and S5, and Figure S1) indicated that the matching algorithm improved the covariate balance of the cohort. Age, which exhibited the greatest discrepancy prior to matching, demonstrated comparable means between the two groups post‐matching (stroke group: 65.98 years vs. non‐stroke group: 66.19 years, *p* = 0.672), with its SMD decreasing from 1.100 to 0.016. Furthermore, the SMDs for core clinical comorbidities, including hypertension (SMD decreased from 0.895 to 0.017) and diabetes (SMD decreased from 0.519 to 0.068), were both reduced to below 0.10. Although a statistical difference in racial composition persisted after matching (*p* = 0.009), the degree of imbalance was mitigated compared to baseline (SMD decreased from 0.280 to 0.130), and this value remained well below the predefined 0.25 acceptability threshold. Accordingly, race/ethnicity was retained as an adjustment covariate in all downstream multivariable models to mitigate residual confounding (a regression‐adjustment‐following‐matching approach).

### 3.3 Independent Effects and Dose–Response Trajectories of Dietary Nutrients

Multivariable logistic regression analysis evaluated the independent associations between individual nutrient intakes and odds of stroke (Table 1). In the fully adjusted model (Model 3), which accounted for demographic and clinical confounders, participants in the highest intake quartile (Q4) for vitamin A, vitamin C, vitamin E, β‐carotene, and selenium had significantly lower odds of stroke compared to those in the lowest quartile (Q1). Importantly, these significant inverse associations remained robust across their corresponding trend tests and Q4‐versus‐Q1 contrasts even after adjusting for multiple comparisons using the FDR procedure (all *q* < 0.05; Table S6).

**TABLE 1:** Associations between dietary intake of antioxidants and trace elements and odds of stroke.

Characteristics	Model1	P1	Model2	P2	Model3	P3
Vitamin A	1.00 (1.00, 1.00)	0.1529	1.00 (1.00, 1.00)	0.2663	1.00 (1.00, 1.00)	0.2155
Quartile						
Q1	Ref	Ref	Ref	Ref	Ref	Ref
Q2	0.88 (0.66, 1.16)	0.3505	0.93 (0.69, 1.24)	0.6025	0.92 (0.69, 1.23)	0.5804
Q3	0.75 (0.54, 1.05)	0.0956	0.80 (0.58, 1.12)	0.1936	0.79 (0.57, 1.11)	0.1729
Q4	0.64 (0.47, 0.87)	0.005	0.69 (0.50, 0.95)	0.0241	0.68 (0.50, 0.94)	0.0193
P for trend		0.0037		0.0157		0.0124
Vitamin C	1.00 (1.00, 1.00)	0.4019	1.00 (1.00, 1.00)	0.6396	1.00 (1.00, 1.00)	0.6142
Quartile						
Q1	Ref	Ref	Ref	Ref	Ref	Ref
Q2	0.78 (0.59, 1.03)	0.0834	0.79 (0.59, 1.05)	0.1097	0.78 (0.59, 1.03)	0.0786
Q3	0.55 (0.43, 0.72)	< 0.001	0.58 (0.45, 0.76)	< 0.001	0.57 (0.44, 0.75)	< 0.001
Q4	0.62 (0.46, 0.83)	0.0014	0.65 (0.49, 0.88)	0.0052	0.64 (0.48, 0.86)	0.0033
P for trend		0.0013		0.006		0.0045
Vitamin E	0.96 (0.94, 0.98)	< 0.001	0.96 (0.94, 0.99)	0.0023	0.96 (0.94, 0.99)	0.0021
Quartile						
Q1	Ref	Ref	Ref	Ref	Ref	Ref
Q2	0.93 (0.70, 1.23)	0.5946	0.98 (0.73, 1.32)	0.909	0.99 (0.74, 1.33)	0.9592
Q3	0.82 (0.62, 1.07)	0.1449	0.88 (0.66, 1.18)	0.396	0.89 (0.67, 1.19)	0.4199
Q4	0.60 (0.44, 0.82)	0.0019	0.66 (0.47, 0.93)	0.0174	0.66 (0.48, 0.93)	0.0167
P for trend		< 0.001		0.0067		0.006
β‐carotene	1.00 (1.00, 1.00)	0.1871	1.00 (1.00, 1.00)	0.2743	1.00 (1.00, 1.00)	0.2382
Quartile						
Q1	Ref	Ref	Ref	Ref	Ref	Ref
Q2	0.67 (0.51, 0.89)	0.0053	0.70 (0.53, 0.92)	0.0113	0.68 (0.51, 0.90)	0.0076
Q3	0.47 (0.36, 0.62)	< 0.001	0.50 (0.38, 0.67)	< 0.001	0.49 (0.37, 0.66)	< 0.001
Q4	0.56 (0.43, 0.74)	< 0.001	0.60 (0.45, 0.79)	< 0.001	0.58 (0.44, 0.77)	< 0.001
P for trend		0.0089		0.0306		0.0252
Selenium	1.00 (0.99, 1.00)	0.0014	1.00 (0.99, 1.00)	0.0064	1.00 (1.00, 1.00)	0.0068
Quartile						
Q1	Ref	Ref	Ref	Ref	Ref	Ref
Q2	0.53 (0.40, 0.70)	< 0.001	0.55 (0.42, 0.72)	< 0.001	0.54 (0.41, 0.71)	< 0.001
Q3	0.59 (0.46, 0.77)	< 0.001	0.62 (0.48, 0.82)	< 0.001	0.62 (0.47, 0.81)	< 0.001
Q4	0.53 (0.40, 0.71)	< 0.001	0.57 (0.42, 0.77)	< 0.001	0.56 (0.41, 0.77)	< 0.001
P for trend		< 0.001		0.0045		0.0049
Zinc	0.98 (0.97, 1.00)	0.0319	0.99 (0.97, 1.00)	0.0855	0.99 (0.97, 1.00)	0.0892
Quartile						
Q1	Ref	Ref	Ref	Ref	Ref	Ref
Q2	0.72 (0.55, 0.96)	0.0271	0.75 (0.56, 0.99)	0.0407	0.74 (0.56, 0.98)	0.0347
Q3	0.84 (0.64, 1.10)	0.2066	0.90 (0.68, 1.17)	0.4233	0.89 (0.68, 1.17)	0.405
Q4	0.73 (0.56, 0.96)	0.0245	0.81 (0.61, 1.06)	0.1261	0.80 (0.61, 1.06)	0.1256
P for trend		0.0877		0.3468		0.3503

*Note:* Data are presented as odds ratios (ORs) and 95% confidence intervals (CIs) for both continuous variables (per 1 μg/day increment for vitamin A, β‐carotene, and selenium; per 1 mg/day increment for vitamin C, vitamin E, and zinc) and quartiles (Q1–Q4) of the six nutrients. Model 1 is the crude model. Model 2 is adjusted for demographic variables. Model 3 is the fully adjusted model, additionally incorporating socioeconomic, lifestyle, and clinical covariates. Owing to the small unit increments, some continuous estimates round to 1.00 at two decimal places; the corresponding *p* values should be consulted for inference. The *p* values in this table were not adjusted for multiple comparisons; the quartile‐specific and trend findings should therefore be interpreted as exploratory. FDR‐corrected results are provided in Table S6.

Zinc intake did not demonstrate a significant independent inverse association (Q4 vs. Q1: OR = 0.80, 95% CI: 0.61–1.06, *p* = 0.126; *P* for trend = 0.350). Restricted cubic spline analyses (Figure S2) indicated non‐linear associations for selenium (*P* for non‐linearity = 0.013) and β‐carotene (*P* for non‐linearity = 0.001). Two‐piecewise logistic regression identified an approximate slope‐change point of 79.8 μg/day for selenium. The inverse association attenuated above this intake, although the post‐breakpoint slope remained weakly negative. For β‐carotene, the estimated breakpoint was 0.87 mg/day, after which the slope was no longer statistically significant. The fitted RCS curve reached approximately 90% of its total decline by 3.0 mg/day, indicating a broader plateau across the higher‐intake range. In an additional sensitivity analysis further adjusting for total energy intake, the inverse associations for vitamin A, selenium, and β‐carotene remained statistically significant (*p* < 0.05), whereas the inverse associations for vitamins C and E attenuated to marginal significance (Table S7).

### 3.4 Joint Effects and Weight Allocation of Nutrient Mixed Exposure on Stroke

Moderate correlations remained among several nutrients in the matched sample (Figure S3), including selenium with zinc (*r* = 0.66) and β‐carotene with vitamin A (*r* = 0.62). These correlations may destabilize the coefficients in traditional mutually adjusted single‐nutrient models; mixture models are therefore warranted. The WQS and qgcomp models were used to evaluate the joint exposure effects of the six antioxidants and trace elements (Table 2). Both models consistently demonstrated a significant inverse association between the overall mixed nutrient exposure and odds of stroke: each quantile increment in the mixed exposure index was associated with 23.2% lower odds of stroke (OR = 0.768, 95% CI: 0.679–0.870, *p* < 0.001) in the WQS model and 26.2% lower odds (OR = 0.738, 95% CI: 0.665–0.819, *p* < 0.001) in the qgcomp model. A formal directionality assessment corroborated the prespecified inverse constraint of the WQS index (Figure S4).

**TABLE 2:** Joint effects of mixed nutrient exposure on stroke evaluated by WQS and qgcomp models.

Method	OR (95% CI)	*p*
WQS	0.768 (0.679–0.870)	< 0.001
qgcomp	0.738 (0.665–0.819)	< 0.001

The WQS model (Figure 2a) revealed that selenium and β‐carotene received the largest weights in the overall inverse association; the qgcomp model (Figure 2b), which imposes no directional constraints, likewise assigned the largest negative weights to these two nutrients. Collectively, these findings indicate that selenium and β‐carotene were the largest contributors to the observed inverse mixture association. In sensitivity analyses additionally adjusting for total energy intake, the weight distributions of the WQS and qgcomp models remained essentially unchanged, with selenium and β‐carotene still contributing the largest weights (Figure S5). The joint inverse association likewise persisted after additional adjustment for total energy intake (Table S8).

**FIGURE 2:**
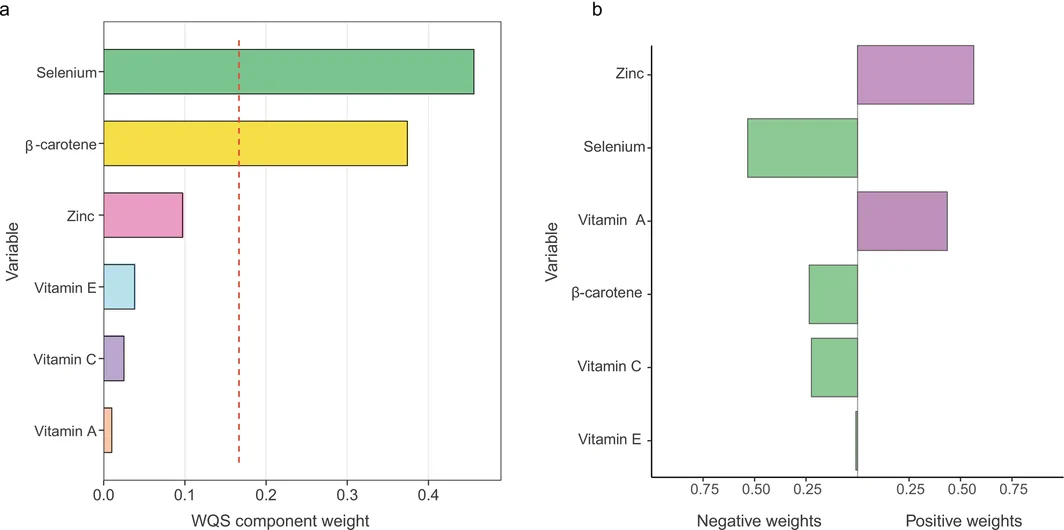
Weight distributions of individual nutrients evaluated by mixed exposure models. (a) Distribution of feature weights negatively associated with stroke in the WQS model. The dashed vertical line indicates the equal‐weight threshold (1/6). (b) Distribution of positive and negative component weights for stroke in the qgcomp model.

### 3.5 BKMR Analysis and Evaluation of Non‐Linear Interactions

A BKMR model was employed to account for the correlated exposure structure among the six nutrients and to flexibly evaluate their joint association with odds of stroke. The overall mixture effect analysis (Figure 3a) demonstrated a significant inverse association with odds of stroke when the joint exposure levels exceeded the median. PIPs (Table S9) identified selenium (PIP = 0.988) and β‐carotene (PIP = 0.975) as the components with the highest posterior inclusion probabilities for this inverse association, whereas the contributions of the remaining components were marginal (PIPs < 0.37).

**FIGURE 3:**
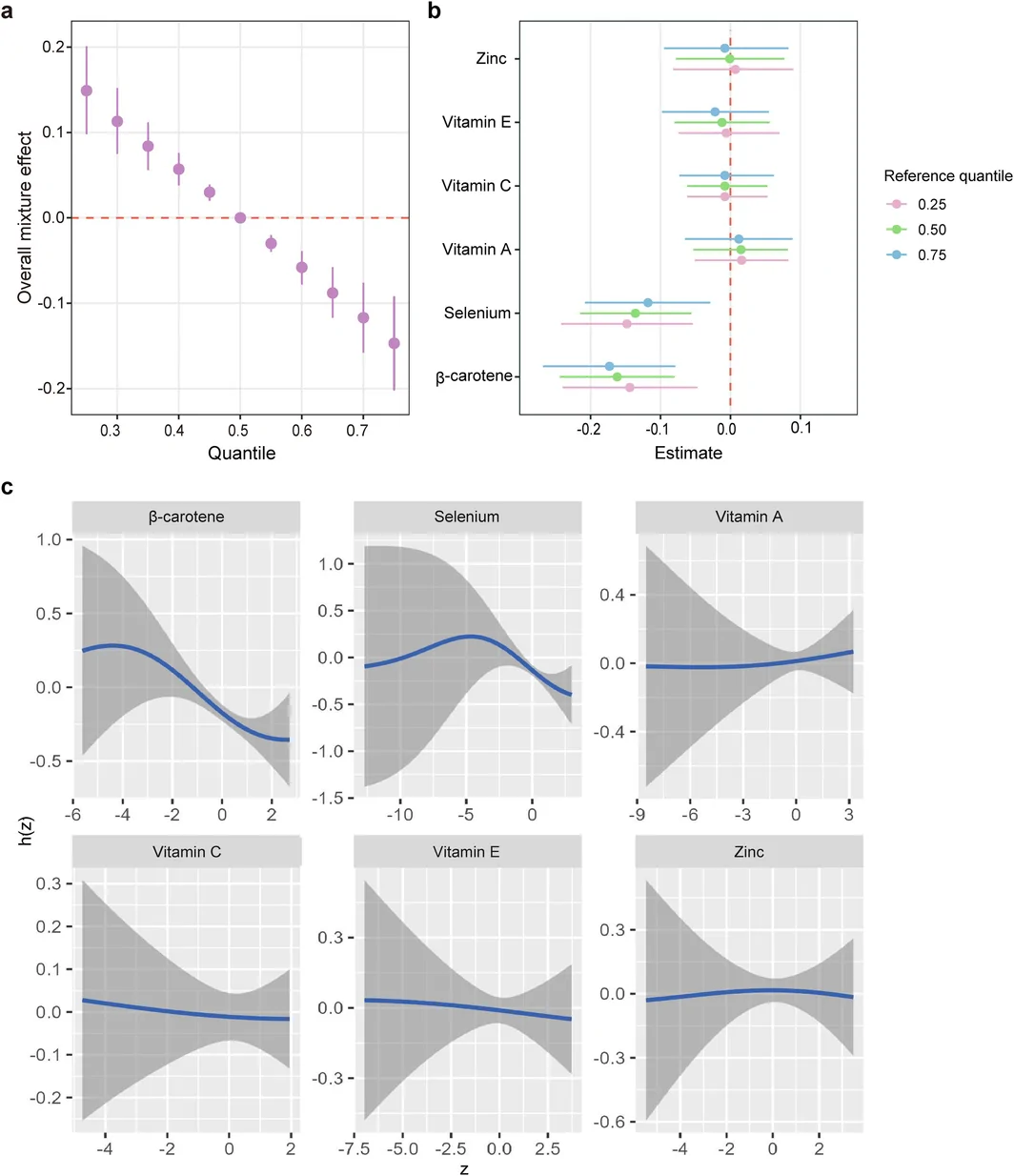
BKMR analysis evaluating the joint and independent effects of dietary nutrients on stroke. (a) Overall mixture effect of the dietary nutrients on stroke, comparing the estimated effect when all exposures are fixed at specific quantiles against their median (50th percentile). (b) Single‐variable effects (estimates and 95% credible intervals) of individual nutrients evaluated at varying reference quantiles (25th, 50th, and 75th percentiles) of the remaining mixture components, assessing potential interactions. (c) Univariate exposure‐response functions for each single nutrient, with the remaining variables held at their median levels. Error bars and shaded bands denote 95% credible intervals.

Interaction assessment (Figure 3b) showed that when the background nutrients were fixed at different quantiles (25th, 50th, and 75th percentiles), the effect estimates and credible intervals of the individual components (particularly selenium and β‐carotene) exhibited a parallel trend, with no statistical evidence of departure from additivity within the investigated intake ranges. Furthermore, the univariate exposure‐response functions (Figure 3c) revealed that, with other variables held at their median levels, selenium and β‐carotene demonstrated a non‐linear inverse association with odds of stroke, characterized by slope attenuation at higher exposure ranges. Conversely, the dose–response curves for the remaining nutrients were relatively flat, indicating no significant independent main effects. The energy‐adjusted BKMR model yielded consistent findings: selenium (PIP = 0.887) and β‐carotene (PIP = 0.991) retained the highest PIPs (Table S9), and the overall mixture effect, single‐variable estimates, and univariate exposure‐response patterns were materially unchanged (Figure S6).

### 3.6 Machine Learning‐Based Feature Importance and Model Interpretation

Four machine learning algorithms (LightGBM, Random Forest, SVM, and XGBoost) were coupled with the SHAP approach to quantify feature contributions. Global feature importance analysis revealed that, across all nutrient‐only models, selenium and β‐carotene consistently ranked as the top two features based on their mean absolute SHAP values (Figure 4a–h). This cross‐algorithm consistency indicates their stable and leading predictive contributions to the model output. Furthermore, SHAP beeswarm plots (Figure 4) demonstrated that high feature values (indicated by yellow points) for selenium and β‐carotene were predominantly clustered along the negative SHAP axis. This pattern indicates that higher intakes of these nutrients are associated with lower odds of stroke.

**FIGURE 4:**
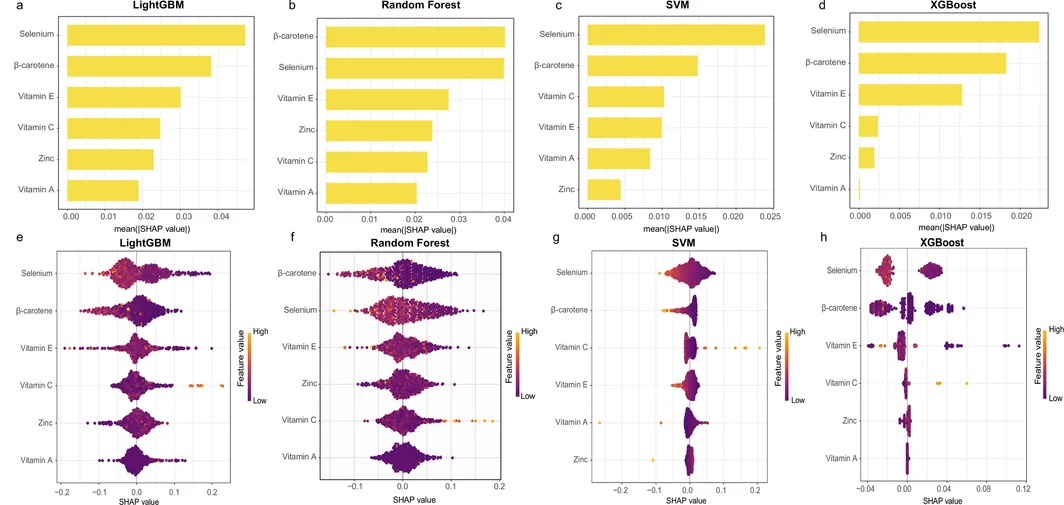
Global and local SHAP‐based feature importance evaluated by four machine learning algorithms. Panels a–d show the global feature‐importance plots (features ranked by mean absolute SHAP value) for the LightGBM (a), Random Forest (b), SVM (c), and XGBoost (d) models; selenium and β‐carotene consistently rank as the top two contributors to the model output across all four algorithms. Panels e–h show the corresponding SHAP beeswarm plots for LightGBM (e), Random Forest (f), SVM (g), and XGBoost (h); each point represents one participant, and point color encodes the original feature value (yellow = high intake, dark purple = low intake). Positive and negative SHAP values denote feature contributions toward higher and lower estimated odds of stroke in the model output.

Notably, in the comprehensive XGBoost model incorporating demographic and clinical covariates (Figure 5a), selenium and β‐carotene retained their positions as the two most important features. Local explanations (Figure 5b) further corroborated the stable association between high exposure to these nutrients and negative SHAP values. Collectively, these findings from non‐parametric algorithms align well with the inferences drawn from the aforementioned regression models (e.g., BKMR), cross‐validating the predominant role of selenium and β‐carotene within this dietary matrix. Details of model discrimination and calibration metrics are provided in Table S10. Across the models, test‐set AUCs were close to 0.5, and Brier scores (0.248–0.252) were close to the value of 0.25 corresponding to an uninformative prediction in this balanced (1:1) test set. Discrimination close to 0.5 is an expected property of the matched design, in which baseline clinical covariates had been balanced between groups and thus contributed little between‐group information for classification; accordingly, the addition of the six nutrients to the clinical covariates did not materially improve discrimination. These metrics should be interpreted in light of the purpose of the machine‐learning analyses—quantifying feature importance rather than developing a clinical prediction tool—as etiologic associations do not necessarily translate into high individual‐level discrimination (Wald et al. 1999; Pepe et al. 2004).

**FIGURE 5:**
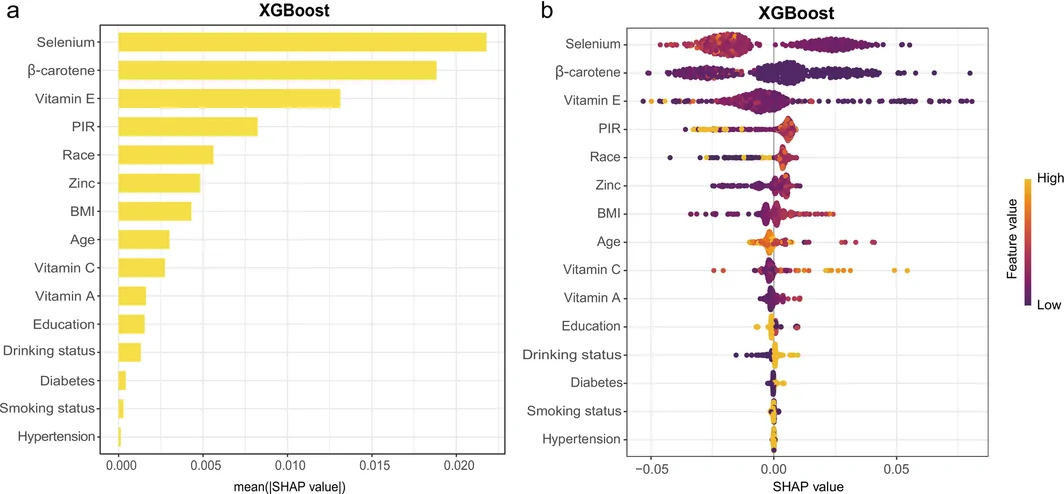
Feature importance and SHAP beeswarm plots derived from the comprehensive XGBoost model incorporating nutrients and clinical/demographic covariates. (a) Global feature‐importance plot, with features ranked by mean absolute SHAP value; selenium and β‐carotene rank as the top two contributors to the model output. (b) SHAP beeswarm plot showing the distribution of individual‐level feature attributions; each point represents one participant, and point color encodes the original feature value (yellow/orange = high values, dark purple = low values). Positive and negative SHAP values denote feature contributions toward higher and lower estimated odds of stroke in the model output.

## 4 Discussion

Previous studies investigating the association between micronutrients and cardio‐cerebrovascular diseases have predominantly relied on single‐nutrient models, largely overlooking the complex multicollinearity and potential synergistic interactions among dietary components (Reedy et al. 2018; Zhao et al. 2021). To address this methodological gap, the present study used a nationally representative large‐sample cohort and first applied 1:1 PSM to balance established baseline confounders, such as age and hypertension.

This design enabled us to examine the joint association between mixed exposure to six dietary antioxidants and trace elements (vitamins A, C, and E, β‐carotene, selenium, and zinc) and the odds of stroke. To further disentangle this nutritional matrix, we subsequently employed WQS regression, qgcomp, and BKMR (Zhao et al. 2021; Keil et al. 2020). The integrated results from these models consistently demonstrated that higher levels of mixed exposure were significantly associated with lower odds of stroke, with selenium and β‐carotene contributing most consistently to the inverse association. Importantly, our sensitivity analysis revealed that while the inverse associations of vitamins C and E observed in the primary models attenuated to marginal significance after additional adjustment for total energy intake, the inverse associations for vitamin A, selenium, and β‐carotene remained statistically significant. This suggests that these associations were not materially confounded by total energy intake. Notably, the consistency of these findings across all models further supports selenium and β‐carotene as the leading contributors to the observed inverse association. In the interaction assessment of the BKMR model, the individual components showed parallel exposure‐response trends without statistical evidence of departure from additivity. Broader clinical evidence supports the value of assessing such combined exposures. Administering high doses of isolated antioxidants outside their natural dietary matrix has not only failed to effectively reduce all‐cause mortality or overall stroke incidence, but under specific intervention strategies (e.g., vitamin E supplementation), such supplementation may even increase the risk of severe adverse events such as hemorrhagic stroke (Jenkins et al. 2018; O'Connor et al. 2022). Conversely, prospective cohort studies of overall dietary antioxidant intake, such as the Swedish Mammography Cohort, have reported that, among individuals without baseline cardiovascular disease, those in the highest quintile of dietary total antioxidant capacity (TAC) had a significantly lower risk of total stroke (Rautiainen et al. 2012). Furthermore, Huang et al. reported a significant linear dose–response association between higher dietary TAC and lower stroke risk (Huang et al. 2024). This “antioxidant paradox” therefore underscores the importance of comprehensive, mixed dietary exposures at the population level.

The biological underpinning of this inverse association relies on an in vivo cross‐phase complementary defense network (Terao 2023). Endogenous antioxidants exhibit distinct spatial heterogeneity: lipophilic β‐carotene accumulates within low‐density lipoprotein (LDL) and cellular lipid bilayers (Terao 2023), whereas the selenium‐dependent glutathione peroxidase (GPx) system localizes in aqueous environments like the cytosol (Ursini and Maiorino 2020). This compartmentalization necessitates functional complementarity. After scavenging lipid‐phase radicals, β‐carotene requires aqueous‐phase reducing systems (e.g., selenium‐dependent enzymes) as electron donors for its regeneration; without this interplay, accumulated pro‐oxidant intermediates could exacerbate vascular injury (Terao 2023). Furthermore, selenium facilitates this cross‐phase cycling and regeneration of antioxidant capacity by dynamically scavenging free radicals and triggering endogenous selenoprotein activation (Xiong et al. 2025). These synergistic mechanisms suggest that primary stroke prevention should prioritize overall dietary patterns rather than overrelying on isolated high‐dose supplements.

Across the analytical frameworks used in this study, selenium carried the largest weight and the most consistent inverse association. Local explanations derived from SHAP values indicated that higher dietary selenium intake was consistently associated with lower odds of stroke. This aligns with the findings of Li et al., who reported that increased circulating selenium levels were associated with a lower incidence of ischemic stroke (relative risk [RR] = 0.88) and lower stroke mortality (RR = 0.86), and that higher dietary selenium intake corresponded to a reduced overall stroke risk (Li et al. 2025). However, the association between selenium and vascular outcomes appears bimodal; excessive intake has been linked to stroke risk factors, such as glucose metabolism disorders and hypertension (Liampas et al. 2023). Notably, our restricted cubic spline (RCS) analysis revealed a curvilinear inverse association with attenuation at higher intake levels, indicating a specific intake threshold. Specifically, the current Recommended Dietary Allowance (RDA) for selenium is 55 μg/day, and the tolerable upper intake level (UL) is 400 μg/day in the United States (Institute of Medicine (US) Panel on Dietary Antioxidants and Related Compounds 2000; Office of Dietary Supplements, National Institutes of Health 2025); the 2023 European Food Safety Authority (EFSA) assessment derived a lower UL of 255 μg/day from selenosis‐related endpoints (EFSA Panel On Nutrition, Novel Foods and Food Allergens (NDA), et al. 2023). In the present study, the inflection point of dietary selenium intake (79.8 μg/day) exceeded the RDA but remained far below these ULs, suggesting that the inverse association operates largely within the range of physiological adequacy, beyond which additional benefit may be limited (EFSA Panel On Nutrition, Novel Foods and Food Allergens (NDA), et al. 2023; Wang et al. 2023).

A similar plateau pattern has been reported for composite antioxidant indices. Although the Composite Dietary Antioxidant Index (CDAI) represents the integrated effect of multiple antioxidants, previous evidence indicates that selenium is an integral component of this matrix (Fan et al. 2025). A meta‐analysis demonstrated that omitting selenium from an antioxidant mixture paradoxically resulted in a significant increase in all‐cause mortality (RR = 1.09, 95% CI: 1.04–1.13) associated with the mixture's intake (Jenkins et al. 2020). This finding suggests that, within a mixed exposure framework, maintaining adequate selenium levels may be important for the potential vascular protective role of the antioxidant network.

The molecular basis of these population‐level inverse associations likely involves the selenoproteome within the central nervous system (CNS) (Huang et al. 2025). During acute stroke injury, heme released from erythrocyte lysis in hemorrhagic lesions, or microscopic iron dysmetabolism induced by local ischemia–reperfusion, leads to severe iron overload and subsequent lipid peroxidation. Although our observational epidemiological design cannot directly elucidate these molecular interactions, prior experimental models provide a robust mechanistic framework. Specifically, this pathological cascade compromises the glutathione peroxidase 4 (GPX4)‐centered defense system, triggering ferroptosis in neurovascular cells (Capps et al. 2026). Experimental models demonstrate that adequate selenium upregulates GPX4, and targeted delivery (e.g., via fusion selenopeptides) can arrest this ferroptotic cascade to facilitate neurological recovery (Capps et al. 2026; Alim et al. 2019). Furthermore, selenium also contributes to the developmental homeostasis of neural circuits and the regulation of post‐stroke complications (Madhwani et al. 2024; Zhuo et al. 2023). Selenium deficiency not only exacerbates acute‐phase injury but also impairs the activity of key antioxidant enzymes, such as thioredoxin reductase (TXNRD) (Liampas et al. 2023), inducing oxidative stress that disrupts the maturation of critical interneurons (e.g., parvalbumin interneurons) and perineuronal nets, ultimately compromising synaptic plasticity and neural network stability (Sasuclark et al. 2025). Importantly, these experimentally observed mechanisms depend heavily on the host's baseline redox status (Rayman 2012). This baseline reliance provides a biological rationale for the fluctuation in health benefits across different subpopulations, emphasizing the importance of considering individual pathological baselines when interpreting observational dietary data for precision nutrition.

β‐carotene emerged as another major contributor, demonstrating a non‐linear inverse association with stroke. This finding not only quantifies the independent statistical contribution of this component to the inverse association with stroke within a complex dietary matrix, but also indicates a distinct dose–response threshold for the potential protective role of the endogenous antioxidant defense system. Specifically, the plateauing trend of β‐carotene (breakpoint at 0.87 mg/day) lies within the range readily achievable through habitual fruit and vegetable consumption. Although no official RDA or UL has been established for β‐carotene (Institute of Medicine (US) Panel on Dietary Antioxidants and Related Compounds 2000; Office of Dietary Supplements, National Institutes of Health 2025), background dietary intake has not been linked to adverse health effects, whereas limiting supplemental use is recommended (Office of Dietary Supplements, National Institutes of Health 2025; EFSA Panel On Nutrition, Novel Foods and Food Allergens (NDA), et al. 2023; EFSA Panel On Nutrition, Novel Foods and Food Allergens (NDA), et al. 2024). Given the cross‐sectional design, these numerical thresholds should be interpreted as hypothesis‐generating reference points rather than prescriptive dietary recommendations.

The inverse associations of β‐carotene identified herein are consistent with findings from multiple large‐scale population studies. A cross‐sectional analysis using the NHANES database revealed a linear inverse association between dietary carotenoid intake and stroke prevalence among older adults, highlighting a significant additive interaction with blood γ‐glutamyl transferase (GGT) levels that exacerbates stroke risk (Zhuo et al. 2025).

Furthermore, the 31‐year prospective Alpha‐Tocopherol, β‐Carotene Cancer Prevention (ATBC) Study cohort demonstrated that higher serum β‐carotene concentrations were associated with significantly lower stroke‐specific and all‐cause mortality (highest vs. lowest quintile hazard ratio [HR] = 0.64 for all‐cause mortality) (Huang et al. 2018). Additionally, moderate dietary intake of β‐carotene has been linked to a lower risk of cardiometabolic multimorbidity (CMM) (Tang et al. 2024); evidence also suggests that total blood carotenoid concentrations below 1000 nmol/L are associated with a higher risk of chronic diseases (Böhm et al. 2021). Although our study identified β‐carotene as a core predictor, null findings from Mendelian randomization (MR) studies imply that elevated circulating levels driven solely by genetic background may not directly translate into clinical protection against stroke (Martens et al. 2021). This divergence indicates that the potential protective effect of β‐carotene more likely stems from a complex endogenous metabolic network, which is substantially influenced by the interaction between individual genetic susceptibility (e.g., BCO2 gene polymorphisms) and overall lifestyle factors (Zhao et al. 2019).

Mechanistically, theoretical models suggest that β‐carotene exerts its antioxidant capacity primarily through the quenching of singlet oxygen and the scavenging of peroxyl radicals (Terao 2023). This antioxidant cascade may delay the formation of oxidized LDL (ox‐LDL), thereby limiting early focal lipid accumulation within the local microenvironment (Jialal et al. 1991). In parallel, β‐carotene alleviates endothelial oxidative stress by shielding endothelium‐derived nitric oxide (NO) from quenching by reactive oxygen species (ROS), a process critical for maintaining vasodilation and endothelial barrier integrity (Abbasian et al. 2023). Furthermore, experimental research suggests that this compound downregulates pro‐inflammatory signaling pathways (e.g., nuclear factor‐κB [NF‐κB]), thereby suppressing the broader inflammatory response (Bai et al. 2005). This anti‐inflammatory effect synergizes with its antioxidant capacity to restrict macrophage recruitment and subsequent foam cell formation, thereby attenuating vascular lesion progression and preserving vascular wall integrity (Gopal et al. 2013). Collectively, although these multi‐target mechanisms are extrapolated from prior experimental evidence rather than directly evaluated in our current analysis, they provide a coherent biological rationale for delaying vascular pathogenesis in primary stroke prevention, consistent with a recent updated review documenting multi‐target protective effects of dietary natural products against ischemic stroke (Zhong et al. 2025).

Although this study leveraged a large‐scale population cohort and employed PSM to rigorously control for baseline confounders, several methodological limitations remain. First, the inherent cross‐sectional design precludes causal inference. Second, a single 24‐h dietary recall primarily reflects short‐term intake and is susceptible to recall bias, and differential reporting according to health status cannot be excluded; thus, future prospective studies integrating serological biomarkers and multi‐omics are warranted. Third, the inability of the NHANES database to distinguish between ischemic and hemorrhagic stroke prevented the evaluation of subtype‐specific heterogeneity. Finally, while 1:1 PSM strengthened internal validity, it inherently compromised external validity. The matching procedure generated a matched sub‐cohort whose stroke prevalence no longer represents that of the original NHANES sample. Consequently, our findings should be strictly interpreted as covariate‐adjusted associations within a controlled epidemiological framework, rather than nationally representative prevalence estimates. Furthermore, this strict matching restricted the natural variation of the sample, meaning the potential for residual effect modification by the matched clinical variables (e.g., age and hypertension) cannot be completely ruled out.

## 5 Conclusion

In this NHANES‐based cross‐sectional study, higher combined dietary intake of six antioxidant‐related nutrients was associated with lower odds of self‐reported physician‐diagnosed stroke. Selenium and β‐carotene contributed most consistently to the observed inverse mixture association across WQS regression, quantile g‐computation, BKMR, and machine learning‐based feature interpretation. The exposure‐response patterns for these nutrients appeared non‐linear, with attenuation of the inverse association at higher intake levels. However, due to the cross‐sectional study design, no causal inferences can be drawn from these findings, and further prospective studies are needed to confirm these observations.

## Author Contributions

**Aifang Zhong:** conceptualization, methodology, writing – review and editing, writing – original draft. **Xinmin Deng:** writing – original draft, methodology. **Yejun Tan:** methodology. **Rui Lai:** validation. **Xiaofeng Lv:** validation. **Xia Zhou:** formal analysis. **Jiaying Zhu:** funding acquisition, conceptualization.

## Funding

This work was supported by the National Natural Science Foundation of China (Grant No. 82260259); the Basic Research Project of the Guizhou Provincial Department of Science and Technology (Grant No. ZK [2023]‐224); the Hospital‐Level Project of Guizhou Provincial People's Hospital (Grant No. GZSYBS [2021]6); the Medical Research Union Fund for High‐quality Health Development of Guizhou Province (Grant No. 2025GZYXKYJJXM0076).

## Conflicts of Interest

The authors declare no conflicts of interest.

## Supporting information


**Figure S1:** Love plot illustrating covariate balance before and after PSM.
**Figure S2:** Non‐linear exposure‐response trajectories of core dietary nutrients and stroke risk.
**Figure S3:** Correlation heatmap illustrating the Pearson correlation coefficients among the six dietary nutrients.
**Figure S4:** Formal directionality assessment of the WQS model in the propensity‐score‐matched cohort (*n* = 2750). Validation‐set odds ratios (ORs) and 95% confidence intervals for the standard negative‐direction WQS model and the positive‐constrained bootstrap‐weight stress test. The standard positive‐direction model yielded no eligible positive bootstrap coefficients (0/1000) and therefore could not produce stable positive‐direction weights. In the stress test, a positive sign constraint was imposed only during bootstrap weight optimization; the final validation‐set coefficient was estimated without a sign constraint and remained inverse.
**Figure S5:** Energy‐adjusted WQS and qgcomp mixture weights in the propensity‐score‐matched cohort (*n* = 2750). (a) WQS weights after additional adjustment for total energy intake; the dashed vertical line indicates the equal‐weight threshold (1/6). (b) Positive‐ and negative‐direction qgcomp component weights after additional adjustment for total energy intake.
**Figure S6:** Energy‐adjusted BKMR analysis in the propensity‐score‐matched cohort (*n* = 2750). (a) Overall mixture effect when all six nutrients were fixed at each joint exposure quantile relative to the median. (b) Change in the latent probit function when each nutrient increased from its 25th to 75th percentile, with the remaining nutrients fixed at the 25th, 50th, or 75th percentile. Points and horizontal lines denote posterior means and 95% credible intervals. (c) Univariate exposure–response functions with the remaining nutrients held at their medians; shaded bands denote 95% credible intervals.
**Table S1:** Unweighted sample sizes by NHANES survey cycle.
**Table S2:** Descriptive comparison of available characteristics between participants included in and excluded from the analytical sample.
**Table S3:** Baseline characteristics of the study population stratified by stroke status.
**Table S4:** Covariate balance analysis of the original cohort before PSM.
**Table S5:** Baseline characteristics of the matched sub‐cohort after 1:1 PSM.
**Table S6:** Nominal and Benjamini–Hochberg‐adjusted *p* values for nutrient‐specific trend tests across Models 1–3 and Q4‐versus‐Q1 contrasts in Model 3.
**Table S7:** Associations between the highest and lowest quartiles of nutrient intake and odds of self‐reported stroke after additional adjustment for total energy intake.
**Table S8:** Joint associations of the six‐nutrient mixture with odds of stroke in WQS and qgcomp models with additional adjustment for total energy intake (kcal/day, continuous), estimated in the propensity‐score‐matched sub‐cohort (*n* = 2750).
**Table S9:** Posterior inclusion probabilities (PIPs) of individual dietary nutrients in the primary and total energy‐adjusted BKMR models.
**Table S10:** Discrimination and calibration performance of the machine learning models in the independent test set.

## Data Availability

The NHANES data analyzed in this study are publicly available at https://www.cdc.gov/nchs/nhanes/index.htm. The analytic code is available from the corresponding author upon reasonable request.
