# Supplementary material for: Dietary Selenium and β‐Carotene Contribute to the Inverse Association Between Antioxidant Mixtures and Stroke: Evidence From NHANES Using Mixture Models and Machine Learning
Source: Food Sci Nutr. 2026 Oct 21;14(10):e72442. doi: 10.1002/fsn3.72442 (PMC13685821; doi:10.1002/fsn3.72442)
Supplement: Supplementary file 1 [file FSN3-14-e72442-s001.docx]

**Supplementary materials**

**Supplementary Table S1. Unweighted sample sizes by NHANES survey cycle**

| **NHANES cycle** | **Total, n** | **No stroke, n** | **Stroke, n** | **Stroke prevalence, %** |
| --- | --- | --- | --- | --- |
| 2001–2002 | 4034 | 3929 | 105 | 2.60 |
| 2003–2004 | 3929 | 3782 | 147 | 3.74 |
| 2005–2006 | 4024 | 3887 | 137 | 3.40 |
| 2007–2008 | 4594 | 4411 | 183 | 3.98 |
| 2009–2010 | 4756 | 4587 | 169 | 3.55 |
| 2011–2012 | 4079 | 3920 | 159 | 3.90 |
| 2013–2014 | 4438 | 4298 | 140 | 3.15 |
| 2015–2016 | 4264 | 4106 | 158 | 3.71 |
| 2017–2018 | 3628 | 3450 | 178 | 4.91 |
| **Total** | **37746** | **36370** | **1376** | **3.65** |

*Note: Values are unweighted counts derived from the final analytical dataset. Stroke was defined as self-reported physician-diagnosed stroke without differentiation between ischemic and hemorrhagic subtypes.*

**Supplementary Table S2. Descriptive comparison of available characteristics between participants included in and excluded from the analytical sample.**

| **Characteristic** | **Included, available n (%)** | **Included, value** | **Excluded, available n (%)** | **Excluded, value** | **SMD** | **P value** |
| --- | --- | --- | --- | --- | --- | --- |
| Age, years | 37,746 (100.0%) | 49.30 (18.00) | 63,570 (100.0%) | 20.34 (22.06) | 1.439 | <0.001 |
| Sex | 37,746 (100.0%) |  | 63,570 (100.0%) |  | 0.003 | 0.659 |
| Male |  | 18,554 (49.2%) |  | 31,339 (49.3%) |  |  |
| Female |  | 19,192 (50.8%) |  | 32,231 (50.7%) |  |  |
| Race/ethnicity | 37,746 (100.0%) |  | 63,570 (100.0%) |  | 0.347 | <0.001 |
| Mexican American |  | 6,145 (16.3%) |  | 16,304 (25.6%) |  |  |
| Non-Hispanic White |  | 17,695 (46.9%) |  | 19,737 (31.0%) |  |  |
| Non-Hispanic Black |  | 7,816 (20.7%) |  | 15,828 (24.9%) |  |  |
| Other |  | 6,090 (16.1%) |  | 11,701 (18.4%) |  |  |
| Education | 37,746 (100.0%) |  | 17,215 (27.1%) |  | 0.248 | <0.001 |
| <High school |  | 3,821 (10.1%) |  | 3,017 (17.5%) |  |  |
| High school |  | 5,346 (14.2%) |  | 2,956 (17.2%) |  |  |
| >High school |  | 28,579 (75.7%) |  | 11,242 (65.3%) |  |  |
| PIR | 37,746 (100.0%) | 2.57 (1.62) | 54,374 (85.5%) | 2.06 (1.55) | 0.320 | <0.001 |
| BMI, kg/m² | 37,746 (100.0%) | 29.11 (6.88) | 50,053 (78.7%) | 22.77 (7.00) | 0.914 | <0.001 |
| Smoking status | 37,746 (100.0%) |  | 18,187 (28.6%) |  | 0.102 | <0.001 |
| Never smoker |  | 20,160 (53.4%) |  | 10,636 (58.5%) |  |  |
| Ever smoker |  | 17,586 (46.6%) |  | 7,551 (41.5%) |  |  |
| Drinking status | 37,746 (100.0%) |  | 10,374 (16.3%) |  | 0.147 | <0.001 |
| No |  | 12,576 (33.3%) |  | 4,190 (40.4%) |  |  |
| Yes |  | 25,170 (66.7%) |  | 6,184 (59.6%) |  |  |
| Hypertension | 37,746 (100.0%) |  | 41,379 (65.1%) |  | 0.572 | <0.001 |
| No |  | 21,345 (56.5%) |  | 33,903 (81.9%) |  |  |
| Yes |  | 16,401 (43.5%) |  | 7,476 (18.1%) |  |  |
| Diabetes | 37,746 (100.0%) |  | 59,003 (92.8%) |  | 0.350 | <0.001 |
| No |  | 32,206 (85.3%) |  | 56,340 (95.5%) |  |  |
| Yes |  | 5,540 (14.7%) |  | 2,663 (4.5%) |  |  |
| Total energy intake, kcal/day | 37,746 (100.0%) | 2128.08 (1007.43) | 50,215 (79.0%) | 1888.01 (941.30) | 0.246 | <0.001 |

Notes: Values are mean (SD) for continuous variables and n (%) for categorical variables; percentages are calculated among participants with observed data for the corresponding characteristic. Available n (%) denotes the number and proportion of each group with observed data. The excluded group comprises all 63,570 participants excluded at any stage from the initial cohort, including 46,316 with missing or indeterminate stroke history. P values were calculated using two-sided Welch's t tests for continuous variables and Pearson's chi-square tests for categorical variables. SMDs are unweighted and were calculated using variable-specific non-missing observations; generalized SMDs are shown for multicategory variables. Absolute SMDs ≥ 0.10 indicate potentially meaningful imbalance. Given the large sample size, SMDs should be used to judge the magnitude of differences, whereas P values are secondary. Education was assessed in adults aged ≥20 years, and total energy intake was derived from the first-day 24-hour dietary recall. Hypertension was defined consistently with Supplementary Tables S3-S5 as mean systolic blood pressure ≥ 140 mmHg, mean diastolic blood pressure ≥90 mmHg, or self-reported physician diagnosis of hypertension (BPQ020).

Abbreviations: BMI, body mass index; PIR, family income-to-poverty ratio; SD, standard deviation; SMD, standardized mean difference.

**Supplementary Table S3. Baseline characteristics of the study population stratified by stroke status.**

| Variables | **Total** | **non-stroke** | **stroke** | **p-value** |
| --- | --- | --- | --- | --- |
| Age | 49(34,64) | 48(33,63) | 68(58,78) | <0.001 |
| Gender |  |  |  | 0.319 |
| male | 18,554(49.15%) | 17,859(49.10%) | 695(50.51%) |  |
| female | 19,192(50.85%) | 18,511(50.90%) | 681(49.49%) |  |
| PIR | 2.19(1.16,4.14) | 2.23(1.16,4.19) | 1.61(1.02,2.86) | <0.001 |
| Race |  |  |  | <0.001 |
| Mexican American | 6,145(16.28%) | 6,011(16.53%) | 134(9.74%) |  |
| Non-Hispanic White | 17,695(46.88%) | 16,966(46.65%) | 729(52.98%) |  |
| Non-Hispanic Black | 7,816(20.71%) | 7,456(20.50%) | 360(26.16%) |  |
| Other Race | 6,090(16.13%) | 5,937(16.32%) | 153(11.12%) |  |
| Education |  |  |  | <0.001 |
| <High school | 3,821(10.12%) | 3,609(9.92%) | 212(15.41%) |  |
| Completed high school | 5,346(14.16%) | 5,081(13.97%) | 265(19.26%) |  |
| >High school | 28,579(75.71%) | 27,680(76.11%) | 899(65.33%) |  |
| BMI | 28(24,32) | 28(24,32) | 29(25,33) | <0.001 |
| Smoking status |  |  |  | <0.001 |
| Never smoker | 20,160(53.41%) | 19,639(54.00%) | 521(37.86%) |  |
| Ever smoker | 17,586(46.59%) | 16,731(46.00%) | 855(62.14%) |  |
| Drinking status |  |  |  | <0.001 |
| No | 12,576(33.32%) | 12,040(33.10%) | 536(38.95%) |  |
| Yes | 25,170(66.68%) | 24,330(66.90%) | 840(61.05%) |  |
| Hypertension |  |  |  | <0.001 |
| No | 21,345(56.55%) | 21,093(58.00%) | 252(18.31%) |  |
| Yes | 16,401(43.45%) | 15,277(42.00%) | 1,124(81.69%) |  |
| Diabetes |  |  |  | <0.001 |
| No | 32,206(85.32%) | 31,319(86.11%) | 887(64.46%) |  |
| Yes | 5,540(14.68%) | 5,051(13.89%) | 489(35.54%) |  |
| Vitamin A, μg/day | 465(253,771) | 467(254,772) | 423(230,731) | <0.001 |
| Vitamin C, mg/day | 54(22,118) | 54(22,119) | 44(18,104) | <0.001 |
| Vitamin E, mg/day | 6.4(4.1,9.8) | 6.4(4.1,9.8) | 5.4(3.3,8.4) | <0.001 |
| β-carotene, μg/day | 730(278,2,230) | 735(281,2,240) | 588(208,1,908) | <0.001 |
| Selenium, μg/day | 99(69,139) | 100(69,140) | 82(56,117) | <0.001 |
| Zinc, mg/day | 10(7,14) | 10(7,14) | 8(5,12) | <0.001 |

Notes: Values are median (interquartile range) for continuous variables and n (%) for categorical variables. P values were calculated using the Wilcoxon rank-sum test for continuous variables and Pearson's chi-square test for categorical variables.

**Supplementary Table S4. Covariate balance analysis of the original cohort before PSM.**

| **Variables** | non-stroke | stroke | p | SMD |
| --- | --- | --- | --- | --- |
|  | 36370 | 1376 |  |  |
| Age | 48.67 (17.85) | 65.99 (13.32) | <0.001 | 1.1 |
| Gender (female) | 18511 (50.9) | 681 (49.5) | 0.319 | 0.028 |
| Race |  |  | <0.001 | 0.28 |
| Mexican American | 6011 (16.5) | 134 (9.7) |  |  |
| Non-Hispanic White | 16966 (46.6) | 729 (53.0) |  |  |
| Non-Hispanic Black | 7456 (20.5) | 360 (26.2) |  |  |
| Other Race | 5937 (16.3) | 153 (11.1) |  |  |
| Education |  |  | <0.001 | 0.24 |
| <High school | 3609 (9.9) | 212 (15.4) |  |  |
| Completed high school | 5081 (14.0) | 265 (19.3) |  |  |
| >High school | 27680 (76.1) | 899 (65.3) |  |  |
| PIR | 2.59 (1.63) | 2.07 (1.39) | <0.001 | 0.344 |
| BMI | 29.08 (6.88) | 29.76 (6.70) | <0.001 | 0.099 |
| Smoking status(Ever smoker) | 16731 (46.0) | 855 (62.1) | <0.001 | 0.328 |
| Drinking status(Yes) | 24330 (66.9) | 840 (61.0) | <0.001 | 0.122 |
| Hypertension(Yes) | 15277 (42.0) | 1124 (81.7) | <0.001 | 0.895 |
| Diabetes | 5051 (13.9) | 489 (35.5) | <0.001 | 0.519 |

**Supplementary Table S5. Baseline characteristics of the matched sub-cohort after 1:1 PSM.**

| **Variables** | non-stroke | stroke | p | SMD |
| --- | --- | --- | --- | --- |
|  | 1375 | 1375 |  |  |
| Age | 66.19 (12.98) | 65.98 (13.32) | 0.672 | 0.016 |
| Gender(female) | 705 (51.3) | 681 (49.5) | 0.38 | 0.035 |
| Race |  |  | 0.009 | 0.13 |
| Mexican American | 164 (11.9) | 134 (9.7) |  |  |
| Non-Hispanic White | 698 (50.8) | 729 (53.0) |  |  |
| Non-Hispanic Black | 318 (23.1) | 360 (26.2) |  |  |
| Other Race | 195 (14.2) | 152 (11.1) |  |  |
| Education |  |  | 0.342 | 0.056 |
| <High school | 202 (14.7) | 212 (15.4) |  |  |
| Completed high school | 240 (17.5) | 265 (19.3) |  |  |
| >High school | 933 (67.9) | 898 (65.3) |  |  |
| PIR | 2.16 (1.46) | 2.07 (1.39) | 0.107 | 0.062 |
| BMI | 29.68 (6.60) | 29.76 (6.70) | 0.774 | 0.011 |
| Smoking status(Ever smoker) | 856 (62.3) | 854 (62.1) | 0.969 | 0.003 |
| Drinking status(Yes) | 811 (59.0) | 839 (61.0) | 0.293 | 0.042 |
| Hypertension(Yes) | 1132 (82.3) | 1123 (81.7) | 0.691 | 0.017 |
| Diabetes | 444 (32.3) | 488 (35.5) | 0.083 | 0.068 |

**Supplementary Table S6. Nominal and Benjamini–Hochberg-adjusted P values for nutrient-specific trend tests across Models 1–3 and Q4-versus-Q1 contrasts in Model 3.**

| Nutrient | Model 1 P / q | Model 2 P / q | Model 3 P / q | Q4 vs Q1 (M3) P / q |
| --- | --- | --- | --- | --- |
| Vitamin A | 0.0037 / 0.012 | 0.0157 / 0.0217 | 0.0124 / 0.0186 | 0.0193 / 0.0232 |
| Vitamin C | 0.0013 / 0.0078 | 0.006 / 0.012 | 0.0045 / 0.012 | 0.0033 / 0.0066 |
| Vitamin E | 7×10⁻⁴ / 0.0063 | 0.0067 / 0.0121 | 0.006 / 0.012 | 0.0167 / 0.0232 |
| β-carotene | 0.0089 / 0.0146 | 0.0306 / 0.0367 | 0.0252 / 0.0324 | 3×10⁻⁴ / 0.0012 |
| Selenium | 4×10⁻⁴ / 0.0063 | 0.0045 / 0.012 | 0.0049 / 0.012 | 4×10⁻⁴ / 0.0012 |
| Zinc | 0.0877 / 0.0987 | 0.3468 / 0.3503 | 0.3503 / 0.3503 | 0.1256 / 0.1256 |

Notes: q values are Benjamini–Hochberg-adjusted P values. The test families were defined as all 18 trend tests (6 nutrients × 3 models) and, separately, the 6 Q4-versus-Q1 contrasts in Model 3.

**Supplementary Table S7. Associations between the highest and lowest quartiles of nutrient intake and odds of self-reported stroke after additional adjustment for total energy intake.**

| Variables | Q4 vs Q1 OR (95% CI) | P |
| --- | --- | --- |
| Vitamin A | 0.76 (0.60–0.97) | 0.025 |
| Vitamin C | 0.80 (0.64–1.00) | 0.050 |
| Vitamin E | 0.77 (0.59–1.00) | 0.054 |
| β-carotene | 0.61 (0.48–0.76) | <0.001 |
| Selenium | 0.58 (0.44–0.77) | <0.001 |
| Zinc | 0.79 (0.61–1.04) | 0.097 |

**Supplementary Table S8. Joint associations of the six-nutrient mixture with odds of stroke in WQS and qgcomp models with additional adjustment for total energy intake (kcal/day, continuous), estimated in the propensity-score-matched sub-cohort (n = 2,750).**

| Method | OR (95% CI) | *P* |
| --- | --- | --- |
| WQS | 0.727 (0.622-0.850) | <0.001 |
| qgcomp | 0.729 (0.634-0.839) | <0.001 |

Abbreviations: WQS, weighted quantile sum; qgcomp, quantile g-computation; OR, odds ratio; CI, confidence interval.

**Supplementary Table S9. Posterior inclusion probabilities (PIPs) of individual dietary nutrients in the primary and total energy-adjusted BKMR models.**

| **Nutrient** | **Primary BKMR PIP** | **Energy-adjusted BKMR PIP** |
| --- | --- | --- |
| Vitamin A | 0.366 | 0.218 |
| Vitamin C | 0.282 | 0.187 |
| Vitamin E | 0.333 | 0.249 |
| Selenium | 0.988 | 0.887 |
| β-carotene | 0.975 | 0.991 |
| Zinc | 0.351 | 0.263 |

Values are posterior inclusion probabilities. The primary model retained the original covariate set; the sensitivity model additionally adjusted for total energy intake (kcal/day). The energy-adjusted estimates were obtained from four chains of 12,000 iterations using the final 5,000 draws from each chain.

**Supplementary Table S10. Discrimination and calibration performance of the machine learning models in the independent test set.**

| **Model** | **AUC (95% CI)** | **Accuracy** | **Sensitivity** | **Specificity** | **F1 score** | **Brier score (95% CI)** | **Calibration slope** |
| --- | --- | --- | --- | --- | --- | --- | --- |
| LightGBM | 0.519 (0.479–0.556) | 0.519 | 0.528 | 0.510 | 0.523 | 0.252 (0.248–0.256) | 0.227 |
| Random Forest | 0.524 (0.487–0.565) | 0.531 | 0.518 | 0.544 | 0.525 | 0.252 (0.247–0.256) | 0.303 |
| XGBoost | 0.574 (0.537–0.614) | 0.564 | 0.741 | 0.386 | 0.630 | 0.248 (0.246–0.250) | 1.829 |
| SVM | 0.521 (0.481–0.555) | 0.508 | 0.487 | 0.529 | 0.498 | 0.251 (0.248–0.255) | 0.275 |
| Comprehensive XGBoost | 0.517 (0.477–0.555) | 0.509 | 0.470 | 0.549 | 0.489 | 0.251 (0.247–0.255) | 0.319 |

**Notes:** Performance was evaluated in the independent 30% test set (n = 825; 413 participants with stroke and 412 without stroke). LightGBM, Random Forest, Support Vector Machine, and XGBoost were nutrient-only models containing vitamin A, vitamin C, vitamin E, β-carotene, selenium, and zinc. The comprehensive XGBoost model additionally included age, sex, race/ethnicity, education, family income-to-poverty ratio, body mass index, smoking status, drinking status, hypertension, and diabetes. Accuracy, sensitivity, specificity, and F1 score were calculated using a predicted-probability threshold of 0.50. The 95% confidence intervals for the AUC and Brier score were estimated using 1,000 bootstrap resamples. Lower Brier scores indicate better overall probabilistic accuracy, and a calibration slope closer to 1 indicates better calibration.

Abbreviations: AUC, area under the receiver operating characteristic curve; CI, confidence interval; SVM, support vector machine

**Supplementary Figure S1. Love plot illustrating covariate balance before and after PSM.**

**Supplementary Figure S2. Non-linear exposure-response trajectories of core dietary nutrients and stroke risk.**

**Supplementary Figure S3.** Correlation heatmap illustrating the Pearson correlation coefficients among the six dietary nutrients.

**Supplementary Figure S4.** Formal directionality assessment of the WQS model in the propensity-score-matched cohort (n = 2,750). Validation-set odds ratios (ORs) and 95% confidence intervals for the standard negative-direction WQS model and the positive-constrained bootstrap-weight stress test. The standard positive-direction model yielded no eligible positive bootstrap coefficients (0/1,000) and therefore could not produce stable positive-direction weights. In the stress test, a positive sign constraint was imposed only during bootstrap weight optimization; the final validation-set coefficient was estimated without a sign constraint and remained inverse.

**Supplementary Figure S5.** Energy-adjusted WQS and qgcomp mixture weights in the propensity-score-matched cohort (n = 2,750). (a) WQS weights after additional adjustment for total energy intake; the dashed vertical line indicates the equal-weight threshold (1/6). (b) Positive- and negative-direction qgcomp component weights after additional adjustment for total energy intake.

**Supplementary Figure S6.** Energy-adjusted BKMR analysis in the propensity-score-matched cohort (n = 2,750). (a) Overall mixture effect when all six nutrients were fixed at each joint exposure quantile relative to the median. (b) Change in the latent probit function when each nutrient increased from its 25th to 75th percentile, with the remaining nutrients fixed at the 25th, 50th, or 75th percentile. Points and horizontal lines denote posterior means and 95% credible intervals. (c) Univariate exposure–response functions with the remaining nutrients held at their medians; shaded bands denote 95% credible intervals.
